# Emotional labor and emotional capital: An interpretive phenomenological analysis of teachers of English

**DOI:** 10.1371/journal.pone.0283981

**Published:** 2023-04-27

**Authors:** Majid Ghyasi, Nurdan Gurbuz

**Affiliations:** Department of Foreign Language Education, Middle East Technical University, Üniversiteler Mahallesi Dumlupınar Bulvarı No:1, Ankara, Turkey; Lingnan University, HONG KONG

## Abstract

An emotionally charged situation for a teacher of English necessitates hiding certain emotions (emotional labor) though using the experience of the event can enable her to benefit from similar encounters in the future (emotional capital). This study is an attempt to find factors that have contributed to the emergence of emotional labor and then investigate whether teachers can gain capital out of such situations. Using Interpretive Phenomenological Analysis (IPA), the study analyzed the diaries and interview data of three teachers of English who had their reflections about daily class incidents. The main themes emerging from the data revealed the existence of emotional labor which, in some cases, the teachers managed to build upon to gain emotional capital. The study suggests diary keeping, teacher bonding communities, and training in order to have emotionally conscious teachers.

## Introduction

The role of emotions in the procurement of great feats or dismal moments for all of us cannot be denied. Human behavior relies on emotions to move ahead, show agency, intensity and energy [1, 2]. However, when it comes to the field of English language teaching, emotions were not given ground for decades owing to the dominance of cognition and rationality [3]. Challenging these major frameworks in action, emotion established itself as a significant role player in learning and this gave birth to new areas of study, such as motivation and beliefs. The affective filter [4] was among the first propositions to give credence to the importance of emotions in the individual’s language learning process. However, the same importance was not attached to the emotions of teachers. Recent studies have witnessed more focus on teachers’ emotions [5]. More importantly, the studies reject the individuality of emotions and argue that emotions are now shaped and performed in culture and society, therefore, they are in line with collective norms [6]. This means eschewing the functional, rationalistic, and linear perspectives on emotions by adopting a poststructuralist notion. In other words, analysis of emotions experienced by teachers should consider the historical, cultural, and socio-political context as well as interpersonal relationships. Emotions are influenced by how a person relates to others and are not solely based on their own feelings [6].

For teachers of English, the context of work necessitates regulating emotions in order to adapt to emotional conventions or emotional rules-defined as feeling rules. Here, the idea of emotional labor can provide rich data on the emotions of teachers of English at work. Emotional labor investigates how emotions are abused at work, how workers sell their emotions to advance work quality and how they frequently hide their true emotions [7]. Viewed in the backdrop of the feeling rules of an institution, society, and culture, emotional labor cross-examines the feelings of a teacher to unravel the genuineness of emotions and the tense state the teacher goes through due to masking certain sentiments. Important occupational matters have already been investigated in terms of the labor intensiveness of certain emotions, such as job satisfaction [8], work engagement [9], and innovativeness [10]. What characterizes our study is the effort made to get close to the feelings of the teacher after a typical day at work and to reinvent emotionally charged situations to see whether or not the teacher undertakes emotional labor.

Regulating one’s true feelings is affected by the predominant *feel* of an institution generated by the influence of cultural history, and that makes emotions a social phenomenon rather than something limited to bodily dispositions [11, 12]. This feel of the situation is what Bourdieu refers to as the habitus, which is a series of dispositions shaped in society [13]. Emotional norms form the *habitus* of a community and determine the acceptance of emotions not based on rules but norms. Similarly, for teachers of English, their habitus is the result of the feel of their community shaped by cultural history, broad socio-cultural factors, and interpersonal relationships at work. [13] explains that the practice of a person is linked to habitus, field (rules and structures) and capital (resources). The concepts were explained in the context of cultural capital, and the idea of emotional capital was later born with the work of [14], who expanded the idea of capital to include emotional capital. It is a form of capital that can be created when there are connections involving emotions, and it can be accumulated and exchanged with other forms of capital [12]. Although emotional capital in education has been significantly investigated [12, 15, 16, 17], a lack of focus on the emotion of teachers is a noticeable feature of these studies. For teachers, if emotions are benefitted and accumulated rather than being acted upon with little or no understanding, an emotional labor situation can be converted into emotional capital [18]. Being successful in accumulating emotional capital, a teacher can be on the path toward both occupational and psychological improvement [19]. Therefore, in the current study, in addition to determining cases of emotional labor and emotional capital, we also aim at closely tracking the emotional journey of the teachers to see whether the conversion from labor to capital happens.

We felt the need to focus specifically on teachers of English as it is characterized as a highly stressful job [20] with a high amount of emotional labor [21]. We also believe that the unique social and cultural context of the current study (schools and a university in Turkey) can enrich the literature in our understanding of emotional labor and emotional capital. The study can both help teachers gain awareness of the emotional side of their practice and contribute valuable findings to the literature by emphasizing the situational nature of emotions and strategies shared by the teachers here to gain emotional capital. This can nourish the poststructuralist theory by proving that generalizations on emotions should be replaced with consideration of the situation and context in which the emotion is experienced. We aim at finding answers to the following questions.

- What are the main emotions three teachers of English associate with their profession?
- What factors contribute to the development of emotional labor among the participants?
- Can the participants convert their emotional experience into emotional capital?

### Teachers’ emotion and emotional labor

A teacher needs to show certain emotions to establish healthy contact with the students, achieve occupational agendas by showing smiles to the management, and prove to be a sociable member once among colleagues. Previous studies have proved the importance of emotions in defining teacher identity, motivation, well-being, relationships at work, job satisfaction, and burnout [22–24].

However, emotions were not long under study due to the label of the prominence of affective neutrality [25], and the sociological convention that viewed consideration of emotions as pre-modern [6]. [7] and her influential book ‘The Managed Heart: Commercialization of Human Feeling’, was among the forerunners to bring emotional investigations up to the stage for sociological analysis. Using the term emotional labor, she observed that emotions in relation to others, mostly in the workplace, were subjugated to ‘the management of feelings to create a publicly observable facial and bodily display’ (p. 7). She refers to the workers of an airline company who are required to situate their emotions to the demands of important role players in their work life. At work, workers have to regulate their emotions in order to conform to the demands and structures which dictate certain emotions as acceptable. The structure acting as the regulatory body of emotions is called feeling rules.

However, what is purported by feelings rules is challenged by constructionist views. In the view of proponents of constructionism, feeling rules are not broad enough to be restrictive and act as rules of society, and they must have a cultural existence [26, 27]. But what [7] refers to as feeling rules is about the structures of a small community formed through years of practice toward a more tangible aim which in most cases is making money. Gauged against the Marxist perspective, [7] sees emotions at work to be exchanged for money. Here feeling rules can act as restrictive elements since the individual needs to subjugate to certain commonalities in order to contribute their role efficiently in the well-being of the organization. [7] did not specifically refer to teachers, but since teaching is affected by neoliberal expansion, it definitely falls under submission to normative feelings to have some feelings under control and express those which can help the company thrive in the competitive job market. [21] sees teaching as an emotional labor job which can put strain on teachers to the point of their complete withdrawal from the profession. Examples of emotional labor imposed on teachers are found in [28]: Masking emotional states to maintain a positive learning atmosphere, keeping an emotional distance from students through surface acting [7], making attempts to stay positive in order to be sources of motivation for the students, and concealment of some emotions such as anger, disgust, and boredom at times of reform. However, the concept cannot be effortlessly labeled as emotionally laborious, according to [29] who state that some emotions are universal feelings for a teacher (e.g. caring and love), and although they might be aroused in teaching, they do not bear the negativity attached to emotional labor. But involuntary expression of care is shown to be a source of emotional labor [28] which proves the situational nature of the emotion that even positive emotions might be a source of labor. An example of the restrictive feeling rules is elaborated in [30] showing how a teacher has to struggle with unequal power distribution in his college while managing his emotions of vulnerability, and how he has to show some care while he has his doubts about the curriculum. Here we can see the power of the declarations dictated from the above, which act as restrictive frameworks and then necessitate teachers to display certain emotions regardless of their real feelings. In another study [31], we can observe feeling rules in the form of accepted norms which are implied to the teachers as ‘common sensical’ to be followed. It is demonstrated how teachers need to have their emotions under control in behaving toward students to be considered ‘professional’ (p. 143). In addition to normative rules, teachers’ own beliefs can be the originator of emotional labor. [32] have shown that the required behavior that needs expressing certain emotions is in contrast with the values of the teacher, resulting in a crisis of competence and ‘emotional exhaustion’ (p. 276).

### Emotional capital

Peter Bourdieu’s [13, 33, 34] influential categorization of resources into economic, cultural and social laid the groundwork for the emergence of emotional capital. The theory was criticized for neglecting emotional matters of social encounters. That was the initial step for emotions to be considered a resource [14]. It is a resource of emotions which can be accumulated over time and can be exchanged with other types of capital [12]. In [14]’s terms, emotional capital is likened to some skills, knowledge, and relationships idiosyncratic to the individual, which can offer them qualities such as affection, empathy, and patience. The feature of capital dispels the idea of emotions being private and individual but are experienced through social relations, are managed in collective social norms [6], and are constructed through social compositions [35]. As an example, a rather common emotion (e.g. anxiety) experienced by the teacher is not viewed as an emotional reaction only, but something that arises from social, historical, and economic context, as well as its relation to power hegemonies [36].

Emotional capital was initially investigated in the light of a resource for women, child-care studies, and mother’s emotional involvement [14, 17, 37]. It was [12] who promulgated the usefulness of emotional capital in educational settings. He points out that emotions can be viewed as resources that can be observed to track their circulation and can be converted to other forms of capital. Teachers empowered by such a capital have an astute feeling of the ‘prevalent emotion norms’ (p.444) to be able to fight back feelings rules of the institution. The concept of habitus, along with institutional and social structures, can influence the accessibility of the teacher to emotional capital gained from individual emotionally charged situations, which brings emotional well-being for the teacher. While in one encounter, the teacher might be overwhelmed by the tense and stressful situation, another teacher can ‘navigate calmly through emotional storms’ [38, p. 113], and shield themselves against emotional breakdown [39]. [12] further continues that emotions transacted among students and teachers can be converted into cultural and social capital. He exemplifies the point by referring to situations where emotional capital results in more sensitivity towards and more understanding of a specific group of people, which leads to improved social relations. An example study in this regard shows how native teachers, as opposed to non-natives, have benefitted from emotional capital to gain access to professional and social networking opportunities that bring economic capital [36]. The study equates a teacher’s emotional capital as important as ‘linguistic, social and cultural factors that might affect teaching practices’ (p. 461).

Awareness raising training and reflective practices of teachers is the first and an essential step in augmenting the teacher’s emotional capital. The availability of such an asset among teachers in China has proved to be followed by educational benefits, although the study has focused on teachers’ emotions at the time of curricular changes. [39] demonstrates how emotional awareness of EFL teachers of a school in China increases teacher engagement and helps them fight problems such as large classes, unmotivated students and extra limitations in the class. [31] study is an investigation of everyday events which reveals how teachers’ enriched resources through their reflective practices help them turn stressful situations into ordinary events and combat the emergence of emotional labor. [31] investigation is one of the few studies taking account of both emotional labor and emotional capital. However, they attest the saliency of conducting more research in different settings with participants of various profiles. Methodologically, what makes our study different is the use of emotional log which tries to lessen the gap between daily events and reporting time. We will provide more details in the following sections.

## Methodology

The study benefits from human participants, so seeking ethical approval was necessary. It was granted by the METU Applied Ethics Research Center (UEAM) (Turkish name: ODTÜ İnsan Araştırmaları Etik Kurulu (İAEK)) prior to the commencement of data collection.

### Research design

In this study, we are trying to interpret two phenomena known as emotional labor and emotional capital in their ‘natural settings’, and in terms of the ‘meaning people bring to them’ through their ‘lived experiences’ [40, p. 43]. That means gaining explication of some complexity; therefore, we need to hear from individuals who are participants of the phenomena. Narrative techniques of qualitative research design enable us to hear teachers’ voices and to make attempts to include all factors involved in the emergence of descriptions. We have benefitted from the principles of the Interpretive Phenomenological Analysis (IPA) methodological approach which takes into account the ‘rich, detailed, and first-person account’ of the experience of the participants [41, p. 58] as well as researchers’ interpretation [42] of the lived experiences. In line with our poststructuralist framework, which focuses on interactions and events in a specific context and has discursive practices into account, IPA looks for shared attributes and distinctive features and is not looking for similarities out of context expressed in formal attributes [43].

### Participants

In order to gain multiple perspectives on the issue at hand from those who have experienced the phenomenon and to reach experienced teachers who could be able to gain emotional capital [18] purposive sampling was carried out. Three female teachers of English, an Iranian native and two Turkish native teachers showed agreement to take part in the study (Table 1). Since the purpose was to have a close and intensive examination of the experience of the participants in relation to two phenomena, it was decided to have a few participants with an exhaustive exploration of their experiences. Prior to the study and over the data collection period, the first author had frequent contact with the participants to establish a bond and gain some basic familiarity with their responsibilities and their place of work in order to create the intimacy to open up and share stories.

**Table 1:** Demographic information of participants.

Pseudonym	Age	Degree	Years of teaching	Place of work	Responsibilities
**Eda**	35	Master’s in English Language Teaching	12	Private university, Turkey	Teaching, supervising and mentoring new teachers
**Songul**	36	Bachelor’s in English Language and Literature	13	Private school, Turkey	Teaching (G5, G6, G11) and coordinating
**Meryem**	35	Bachelor’s in English Language Teaching	8	Private school, Turkey	Teaching (high school), organizing clubs (extra speaking courses)

As required by the METU Applied Ethics Research Center (UEAM), written consent was obtained before the study began. The informed consent form includes general information about the researchers, purpose of the study, possible benefits and potential risks (if any) to participation, expectations of the researcher, approximate time of the study, assurance of confidentiality, and voluntary basis of the study.

### Data collection

IPA proposes narratives, focus groups, and interviews as data collection tools. Due to criticisms against focus groups which value the voice of the crowd over individuals’ ideas [44], narratives and interviews were employed as our data collection tools. To prepare the participants for the narrative phase, the researchers ran a briefing session personally with each participant to clarify the task of journal keeping. They were asked to keep an emotional log for three weeks during which they would write down the daily events as they happened and describe their feelings and emotions in such situations. Some related questions acting as hints (available in the S1 Appendix) were utilized to help retrieve the emotional experience of the day. These localized questions were adopted from [45] and [21]. To boost the credibility of the data, the participants were granted complete freedom in what they wished to include in their daily narrations [31–41], and were given the option of sending voice messages, even in their native language, instead of completing the journal; an option which was embraced by two of the participants. The stories obtained over the three-week period made the teachers have reflective practice over daily events and enabled the researchers to analyze emotions and decide whether the experience of the teacher falls under the defined phenomena.

The other data collection tool was interviews which were planned to give the teachers moments to talk more about some of the chosen incidents, describe their feelings in more detail and talk about the solutions they had reached. Drawn from the previous studies that had benefited from interviews on teacher emotions, questions were formulated while having the general topic of the study in mind [46]. They were designed to enable the questioner to reach the target question sideways—to have general to specific questions so that the main point is addressed, while the principle of the interviewee’s freedom is not breached [41]. We did not stick to an already set number of questions as the screening of the written narratives determined the number and types of questions. One single piloted interview session ensured the applicability and validity of the questions.

The participants, who had been informed of an interview at the beginning, were invited to the session which happened in two days, each lasted roughly twenty minutes, and one was conducted online. We asked questions such as: ‘How did you feel in this situation?’; ‘Do you think you had to hide some emotions?’; ‘Have you already had incidents of the same kind?’; ‘Was there any take-away for you from this incident?’. The interviewer, the first author, tried his best to lead the conversation toward gaining rich data on the topic, but made the participants speak freely to have the interview session more like a friendly dialog than a structured formal examination. Interviews were audio recorded and transcribed verbatim, and the emotional logs data was converted to textual data for close analysis.

### Data analysis

In order to answer the first research question-the main emotions teachers associated with their job-a categorization proposed by [47] was employed. This includes basic emotion prototype, subordinate categories, and sub-clusters which clearly delineate various types of emotions. This categorization has the following emotions: Love (affection, lust, longing); joy (cheerfulness, zest, contentment, pride, optimism, enthrallment, relief); surprise; anger (irritation, exasperation, rage, disgust, envy, torment); sadness (suffering, sadness, disappointment, shame, neglect, sympathy), and fear (horror, nervousness).

We used iterative and inductive analysis [48] to determine sets of data which can be evidence for the next two research questions, while having our poststructuralist framework as a guideline to do our best to consider all the factors involved in a statement. After line by line analysis of the data, we drafted codes related to each chunk of data which were deemed to be related to the aim of the study. Frequent revisiting of codes gave us the highest compatibility among the codes, and enabled us to form themes based on invariant constituents [49]. The researchers then interpreted the participants’ concerns by trying to see the world of the participant and assessing the viewpoints against the factors which can be counted as emotional labor and emotional capital.

After framing coherence and ensuring plausibility of interpretations, a full narrative was achieved. During the data analysis, some emergent themes were excluded from being analyzed as they would not contribute anything to the aim and scope of the study [41]. The frequent contact of the first author of the present study with the participants over the data collection period, the experience of the researcher as a teacher of English, as well as the familiarity of the second author with the contextual variable (working as an academician in Turkey for over 30 years) played a major role in the coding process and determining the themes and their connections.

**Fig 1:**
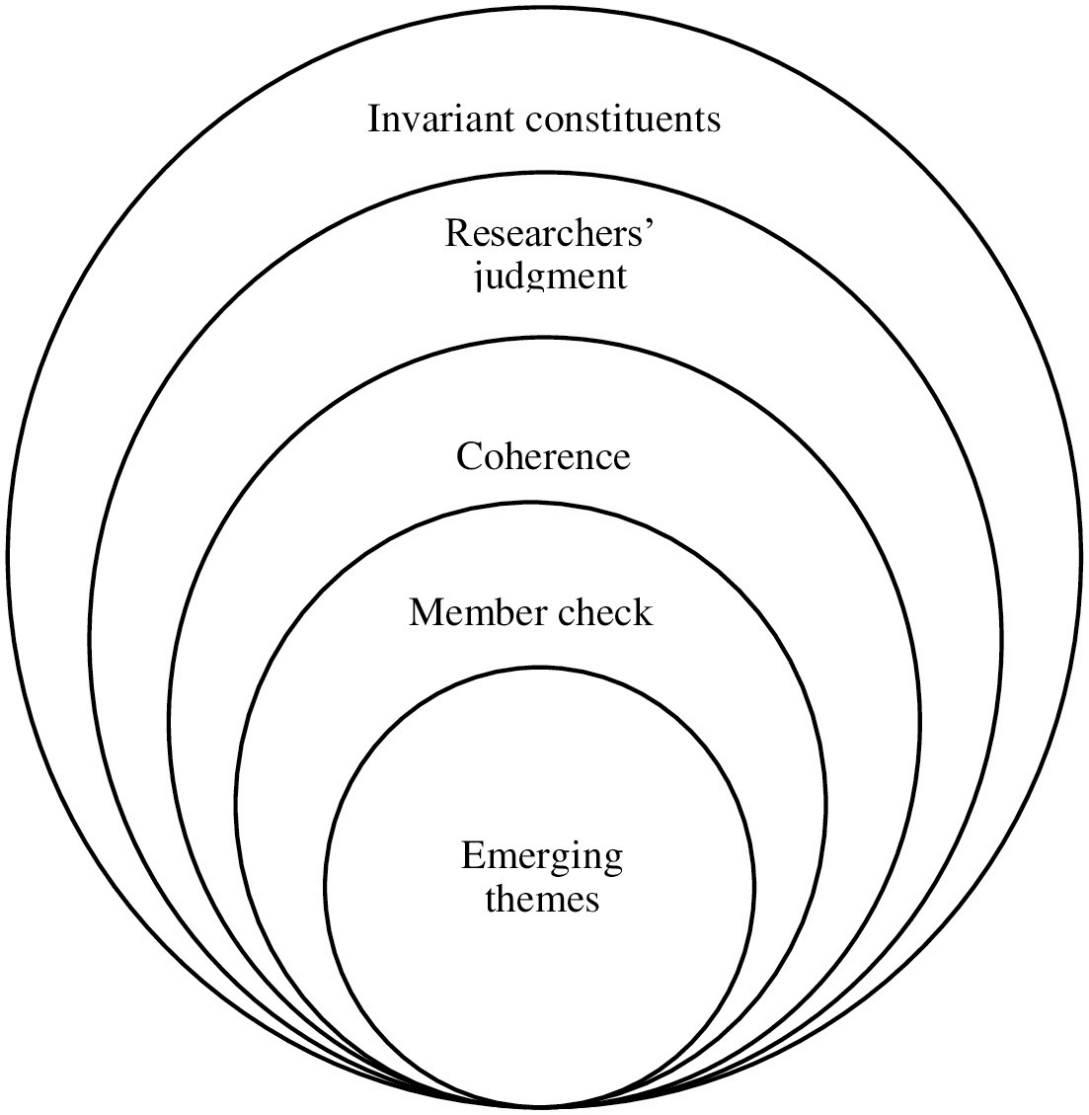
Steps to acquire themes.

The necessity of an agreed-upon decision concerning what to be used for categorization and how to do theme construction can be proof of the trustworthiness and credibility of the data. As the final step, a member check strategy where the participants were informed of our judgment and were consulted about our interpretation of data helped us reach more valid findings. Fig 1 shows the steps taken to yield the major themes discovered from the data obtained from both narratives and interviews.

## Findings

### Teachers’ emotions

Analysis of teachers’ diaries and their interviews helped us to a general understanding of common emotions in their classes. It also provided the ground for a further analysis of emotional labor and emotional capital in this setting. As mentioned before, the taxonomy developed by [47] was used as a reference to categorize the self-described emotional status of the teachers. Our analysis proved the existence of some positive emotions, which we are not going to dwell on more than a quick reference to what the participants said. Love and joy as basic emotion prototypes [47] were reported by the three teachers. Joy is felt by the teacher when she observes her students’ progress in expressing themselves in English. There are also distinctive situations which bring positive feelings for the teacher. For example, Meryem experiences feelings of joy and contentment when she talks about an ordinary day at work when she does not have to express any extreme feelings. Despite moments where she must raise her voice for class management purposes, she downplays the severity of this and has accepted the fact that this is an indispensable part of her job. At the end of the day, she is pleased to have her routine, meaning she does get swayed from the main schedule because of getting involved in showing intense emotions. For Songul, positive feelings arise from her success in creating a friendly classroom atmosphere in which there is a lively but serious discussion in English. She feels proud when her worldview and experience can come to her assistance to have an enlightening conversation with students about an important current event. On the other hand, the analysis of diary entries shows that the school’s negligence in accommodating some basic amenities and failure in having everything planned greatly account for the emergence of negative feelings, more noticeably, anger. Songul feels a lot of anger towards the school’s poor planning and organizing class hours, inefficient equipment, too many teaching hours, and extra imposed responsibility on the teacher such as material development and test preparation. Eda talks about institutional failures, including transport, and tea drinking facilities as sources of anger and frustration for her. Other causes of such intense feelings include low proficiency of her students due to poor placement tests, unreasonable expectations of the students, and high amount of absenteeism on days approaching the end of the semester. As an example, Songul shares her reaction to a simple change in her school.

*The classroom where the class is held changes almost every day*. *I mean*, *I should check it every day*. *And there’s no educational reason behind that unfortunately*. *They just do it so that students won’t get used to one place*, *but it makes me agitated to have another burden of keeping track of class numbers*.

(entry 1)

Songul constantly complains about slight changes in the weekly schedule, such as class hours and classroom numbers, which were supposed to improve the efficiency of the educational program. However, she sees them as an additional burden on the teacher who has to show flexibility and adapt herself to these frequent changes. It can be seen from this data that reform, which can be defined as changes supposed to boost efficiency and profitability, seems to be a source of negative emotion for teachers. As can be observed from Songul’s emotional state, poorly-planned reforms can take its toll on the teacher’s emotional health. Reforms are mostly done to boost the efficiency of education by having everything structured; a phenomenon based on the presence of neoliberal ideologies in education [50, 51]. However, what is observed here is that reform in the school is being carried out without any attention to how teachers feel toward that change.

Another reason for our teachers’ anger, which is deep down due to poor planning, is the misbehavior of the students. Here, we can see how Meryem has to confront some students.

*… today two [students] were playing chess while I was teaching*. *I confiscated the chess set*, *but then I realized they had taken it from my desk drawer and started playing again … another one was singing in the class*… *They do not care at all since they’re at an advanced level*. *The point is I have two periods of lessons with them every day*, *meaning I should see a lot of them every day*. *The problems are temporarily solved when I refer them to the one in charge of discipline*, *but it’s never totally resolved*.

(entry 2)

The unruly behavior shown by the students here can be due to their lack of interest in the lesson, which is mostly the result of mismanagement. The school has not managed to cater well for the language needs of advanced level students. The number of hours is another problem, as the students have to sit in English classes every day. Due to the importance placed on English lessons in almost all private schools in Turkey, syllabus designers do their best to devote a remarkable number of hours to English lessons. Since some students start schooling years with an acceptable level of English proficiency, they pass through these levels more quickly. At advanced levels, these students cannot fit into the typical proficiency-based syllabus, have to repeat some courses and then feel bored in the English class. Meryem constantly refers to the traumatic feeling she has when she even thinks about giving lessons in this class; a situation replete with negative emotions for which the planning and syllabus designing is to blame for its existence.

### Emotional labor

With respect to the poststructuralist view which emphasizes the discursive nature of emotions formed in social relations [52], we have found examples of emotional labor born out of the restrictive rules and norms in the immediate and broader context of work. In another major theme, we can see that these restrictions make the teacher mask the real feeling of care that they have at the moment.

#### Structures and rules being restrictive

Attempts to express unrealistic emotions can be due to rules and structures present in an institution, the breach of which can result in work contract termination. Satisfying feeling rules [52, 53] imposed by various role players who mostly hold managerial roles might seem more important than educational matters. Marketization of education, where efficiency is defined as the number of new enrolments, necessitates formulating rules to achieve the aims of the institute. Everyone involved in the system is supposed to follow rules, sometimes at the expense of ignoring their own individuality and agency. Such a policy introduces a mismatch between the feeling rules of an institute and the feelings of teachers. At the beginning of the data collection period, this is what Songul writes in her diary about her limitations at school.

*… what got on my nerves was a message from the boss asking everyone for a meeting in the last two hours*. *Why was this painful*?! ….. *I found it insulting and nerve-racking*. *We cannot react to this as we are "yes sir*, *boss" at work*. *So I had to swallow it and let it be like before*!

(entry 3)

In Songul’s school, the last two hours are devoted to preparing materials or just relaxing, but sometimes teachers are called to a meeting. Songul does not like to attend meetings after a hard day at work, but she has to swallow her emotions and present herself as a subservient employee. In another part of her diary, she refers to moments when she had to pretend to be working during the last two hours; a sort of revert to some ostentatious behavior to spread the unreal feeling of conscientiousness among the new teachers. In the interview, Songul laments her experience to be to her disadvantage, since that has made her responsible for showing enthusiasm in meetings in order to be a positive influence on young teachers, magnify the authority of the boss, and mirror everything as professional. Not only is the act of hiding some emotions, but also generating an unreal sentiment brings an emotional labor situation. An instigator of emotional labor is to force an employee to produce some negative or positive emotional states in the customer [7]. This is also evident in a diary that Meryem has written about an expectation from her supervisor:

*… our coordinator wants me to find some seminars for students to attend*. *I am not paid extra for having club sessions*, *but she wants me to search for seminars by taking my between-semester holidays*. *I need to motivate students*, *and also check their attendance*, *and all these things make me feel really sorry about this scheduling*.

(entry 4)

The teacher is snowed under material preparation that exerts its emotional burden on the teacher since she believes this is not something good for the students. Disregarding her own stance here, the teacher should produce an emotional expression that is not in agreement with her real feelings; something she believes is derived from poor planning. The feel of her institution has created a norm that any type of disagreement equals a rebellious act, and that these teachers are prone to dismissal at the end of the semester. This has created a huge gap between the teacher’s real feelings and what she showcases. In such an atmosphere, the only thing the teacher can do is to hide her true feelings in order not to be considered a rebel and just succumb to the feeling rules.

In a less customary situation, Songul feels incompetent in infringing on the norms of the school, which she believes such normative behavior are not just limited to the immediate context but the whole society embraces it. That is because the habitus of her place of work is backed up by important people in the lives of the students to keep the status quo.

*…in these situations*, *I cannot interfere*. *It’s what the community wants*. *It’s what their parents want*. *If I want to jump in*, *I might face problems*. *A teacher can’t save the kids… I believe it’s just the school culture*. *I won’t be able to do anything*. *I’d rather walk away*. *It is not just one person*. *It is the dominant ideology*…

(entry 5).

Here the principal has inflicted a severe punishment on all grade-five students because of violation of some disciplinary rules. They are made to stand up for hours doing nothing until they feel exhausted. Observing such harsh treatment, a teacher wants to protest it, and Songul has a reflection about her role here. She accepts the fact that she cannot fight the principal, the parents and all the people who approve of such strict manners, so she just prefers to walk away. The accumulation of dispositions in society prevents Songul from applying her agency and makes her accept the situation as it is. It is not just the habitus of the school, but that of society which stands against her true feelings and forces her to just hide them.

#### Hiding the feeling of care

The other theme emerging from the data shows cases where the teacher is willing to care about her students, but she feels that she should abstain from appearing as a caring teacher as this might be exploited or it could cause problems in the shadow of norms of the community or society at large. Meryem reported experiencing this due to the misbehavior she frequently observed in her class.

*I am generally a kind and caring person*, *and I feel frustrated when they* [the students] *try to take advantage of that …Sometimes I go to the class intentionally mimicking an angry look*. *It is just to show that I am serious*, *so they pay more attention*.

(entry 6)

Meryem tries to have her emotions under control, mask her true self, and present herself as a serious and insensitive person in order to have a more obedient class. This is in spite of the fact that care is universally believed to be positive, but it shows that the type of situation the teacher is involved in determines the emotions that need to be expressed. For Meryem, the label professional is received when she distances herself from her true self, as those teachers in [31] investigation in which the teachers have to bear emotional labor to be seen as professional. In another example below, as an obvious example of emotional labor, what Songul reveals is not compatible with what she really feels.

*The students’ hugging me has created some problems for me*. *There are a couple of students who do not let go of me when they start hugging*. *They keep going and hugging harder for a couple of minutes*. *They’re two boys*, *five graders*. *I try to make them stop and have them sit down*, *but they press on*. *The problem is that it is too much*, *and it takes a lot of time*. *I can’t behave harshly here; they’re just kids showing sentiments*. *I try to convince them with jokes and a lot of smiles to make them get back to their seats*, *though they do it again from time to time*.

(entry 7)

Songul has to fabricate a happy face (surface acting) and express nice feelings to keep the students satisfied. However, her genuine feelings at the interview session showed how stressed she was at hug times although she really was willing to join the love sharing moments. The feeling rules have contradicted the situation and have caused the teacher a great deal of fear. There is the fear of not covering the syllabus and enraging the principal (institutional constraints), and the fact that it might be seen as a rather unconventional act (social constraints). Considering the society where she works, she is not reassured of having complete freedom to act as she wishes when she is hugged by boys many times for a long time. She believes this is an unorthodox scene not accepted in a culture in which there are restrictions when it comes to opposite sex interactions despite students’ very young age. As we can see, broader social norms and cultural factors regulate the way a teacher should show feelings in interactions in the class.

Another instance of hiding care is when Meryem goes through multiple emotionally charged events in just a few minutes of a typical day at work. This shows how she has to fluctuate between different emotions when she weighs up various options to fulfill the wishes of important stakeholders in her career.

…*there was a small mistake in their* [English] *test paper… the students had talked inappropriately to one of my colleagues who had got offended due to the behavior of my students…*. *gesturing a serious face*, *I then stepped into the class and told them*: *‘I don’t care about your proficiency in English*, *it’s your behavior which matters to me’…*[A few minutes later] *I started the lesson trying to be serious*. *Later on I wanted to show them a TED talk*. *As soon as I played the video*, *there was a half-naked girl shown on the screen* [as part of the talk]. *The class burst into laughter and silly comments could be heard here and there… I felt embarrassed and had to bear student’s sneers*…

(entry 8)

It is a challenging situation when she has to keep her friend happy by showing her anger at students because she cares about how her friend feels. At the same time, she needs to adjust her feelings toward what students have gone through because of a mistake in their exam paper, while trying to show respect to test designers. About the video incident, she needs to hide her frustration as she needs to show concern about those material developers. But she also needs to obey the conventions of an Islamic society which does not fully embrace the display of such content in an educational place. In just a few minutes, she has been acting like a savior, guardian and a good member of society; a cascade of emotions which makes her feel that all the things and people she regards as important can be ruined by just one small incident. Meryem told us she was wretched not knowing what should be the priority when she had to show care: Is it her students, her colleagues, or the culture, or not caring at all. As this incident shows, one all-inclusive term cannot define how Meryem feels while she is trying to regulate her emotions in connection with a new case emerging. Immediate constraints as well as socio-cultural factors force the teacher to hide or feel unsure to show one of the most essential emotions for a teacher.

### Emotional capital

This section tries to analyze the diaries and assertions of the three teachers to see whether they have managed to gain any benefit from an emotionally labor intensive situation by enriching their emotional repertoire. In this connection, we have tried to closely follow the teacher’s emotional journey which is affected by institutional, social, cultural, and personal factors shaping the emotions of the teacher.

#### Conversion of emotional capital

By this theme, we mean cases in which the teacher has managed to benefit from having her emotions under control and then succeeded in gaining social, political or even economic capital [12]. Such observance can provide the answer to the third research question mentioned in the introduction. The theme can be exemplified here by referring to the reflective practices that Songul had at times of her encounter with young learners. Songul has been a teacher of English to adults for many years but seeking better job opportunities made her accept some classes with teenagers. In spite of being a rather experienced teacher, Songul struggles to have classes with young learners under control to the extent of yelling at times of anger. Careful interpretation of her diaries in light of emotional capital proposals made us spot situations which have actually helped Songul deal with a stressful situation.

*Something I learned today was that shouting is not that effective*, *but applying some sense of humor* [*can work better*] *…now they like me and I love them too*. *Although I am never ever into teaching kids*, *I have managed to handle these 17 children*.…

(entry 9)

Here, Songul has managed to use emotions as a resource and change them into good relationships with her students. This proves improved social relationships achieved through emotion management. In the interview, she told us that her true desire might be to shout and keep the students completely quiet as that is what her impulsive feelings demands; however, multiple recurrences have proved that the action is not effective. The presence of emotional labor is out of the question, but it can be assigned as the positive version of emotional labor. It is a strategy known to make teachers able to benefit from the management of their emotions [32–54]. Songul states that a noisy and inattentive class engages her mind for hours after the class and even shares her disappointment with her husband to have a strategy to ‘turn the tables’ to her benefit. Her diary entries show further success stories in managing young students. For example, when students are having arguments with each other, she tries not to lose her temper but calmly establishes her role as the person in authority, reminds students of their friendship, and pictures alternatives for the students. She used to lose her temper at the beginning, but in time manages to hide the rage in order to prevent negative feelings from surfacing. The ability to have such a management capability proves the cumulative features of capital. It is obtained once the individual encounters situationally unique cases and then tailors various solutions accomplished through meticulous reflection over the incident and then passionately looks for a remedy. The improved social relations can also be found in the following example:

*Yesterday I had a chaotic class*. *Students love coming to the board without asking permission and they start drawing something…I want to ask them to sit*, *but then I see a heart with my name in it*. *It’s nice but they should sit because it’s just killing time*. *But I can’t get angry at them*. *I try to copy their drawing*, *ask some questions about love*, *and then demand they sit*. *It has worked so far*.

(entry 10)

In this example, for Songul, the situation is a case of a multitude of emotions that can bring emotional ups and downs for the teacher. Songul shows confidence in how she manages to bring the situation under control by avoiding blurting loving back sentiments or giving in to negative feelings. She studies the incident and, with her familiarity with the students, tries to act in a way that leads to the smooth transition to teaching time. The display of positive emotions has always been referred to as a sign of good teacher/competent teacher [55–58]. Nonetheless, we observed that knowledge of human emotions enriching our emotional sources significantly defines the success of the teacher, rather than the display of feelings universally labeled as ‘positive’ or ‘negative’. While the habitus of the school positions Songul at a too caring level, accumulation of social capital has enabled the teacher to change the habitus present in her class. The creation of new affective connections enables the teacher to occupy a new position in the habitus and make changes to her emotional well-being [12].

#### A cause yielding emotional capital

Besides the situational factors, socialization of individuals affected by their educational, social, and cultural background contributes to the accumulation of embodied capital [33, 34]. This means one’s affluence on different types of capital can determine a person’s ability to acquire emotional capital. Under the current theme, what we mean by a cause is a belief or an ideology that the teacher wholeheartedly follows either in their private or professional life. For many, the cause is defined by the person in connection with the strength of different capitals. In her daily diary, Eda explains how she is in control of her emotions.

*Some students don’t come* [to the class] *just because I keep speaking English all the time and they struggle* [to understand]. *I question myself in such situations*. *I try to be consistent and keep a profile based on my teaching philosophy*. *I have some flexibility of course but I shouldn’t push those limits that much as they ask for*.

(entry 11)

Although questioning her own reaction with regards to the students’ expectations, Eda claims that her teaching philosophy has made her feel content and sure about her performance in class. Prevailing over emotions can be feasible by channeling them toward a final aim, which in this case is Eda’s teaching philosophy. In the interview session, Eda mentions that she empathises with her students as she knows their problems, but, after all, prioritizes her values over the demands of the students. This has enabled her to triumph over the odds. She has managed to hide some emotions, but the act can be seen as a resource rather than a problematic situation. As another example, Meryem kept a record of an ordinarily annoying situation that did not cause her to feel insincere about her emotions because she has her own view when it comes to students of a certain age.

[on the bus] *there’s a 7-grade student who does not wear his mask properly*. *I reminded him of that a couple of times*. *Then he removes his mask and yells at me*. *He says*: *‘I won’t wear it*, *and don’t talk to me’*. *I told him I was addressing everyone*. *When getting off*, *he was saying I don’t want to see you again*… *Before we get off the bus*, *our responsibilities are still there like something never ending*.

(entry 12)

Meryem has to accept the responsibility of school bus monitor at the end of almost each school day. She genuinely does not see it as her responsibility but tries to show a nonchalant attitude here. She could have lost her temper at the behavior of the kid, but she showed warmth and gentleness. In the interview session, Meryem graciously says that they are just kids, and that she does not hold grudges against them. The situation could have turned to resentment and anger if she had to show some emotions in order to comply with the rules of the school. However, it is considered a case of emotional capital because she gauges the situation against her belief and makes a decision sourced from that belief.

## Discussion

The study explored the self-reported emotions of three teachers of English with a focus on instances of emotional labor and acquisition of emotional capital. We found feelings of joy and affection coming from the relationship the teachers have with their students. However, mainly due to poor planning in the institution and not providing basic facilities, teachers feel unappreciated and angry. Students’ disobedience, mainly derived from mismanagement, is another reason for our teachers to experience negative feelings.

The findings disclosed situations in which our teachers had to cover up their true feelings; cases which were regarded as emotional labor. At some points, they have to refrain from showing the caring feeling that they feel like expending at the moment. While previous studies place emphasis on teacher’s care, here we expand the proposal made by [29] who assert that appearing as a caring teacher necessitates a significant amount of emotional labor. Not only is overvaluing some emotions like caring for an instance of emotional labor, but also hiding this feeling means downplaying emotions, thus an emotional labor situation.

One of the main restrictions on teachers which results in emotional labor is the feeling rules specified in the forms of rules and managerial expectations. We showed how Meryem’s principal wants her to be motivating for students regardless of her thoughts and feelings about that issue. We do not intend to reject the positive role of faking enthusiasm in students’ affect and their educational outcomes (see [59, 60]). However, what happened to Meryem is an instance of emotional labor wherein the teacher has to bear the motivational burden [20] and experience emotional distancing which in the long run can lead to ‘emotional exhaustion’ [20, p. 103]. Spreading a motivating vibe may lead to positive results for the students; however, the teacher’s unmotivated emotion covered with enthusiastic generative smiles can turn the teacher into a life-long actor, and drops her real self into a happy teacher on duty and a sad teacher off duty.

In other cases, the entries recorded by Songul about rules in her school clearly show that there are emotional filters active in her workplace which coerce the teacher to abide by certain emotional rules [7] and cap the expression of certain emotions. She believes that is what the institute wants them to be like, so she prefers to avoid any confrontation. As such, the institutional feeling rules here are the disposition to have obedient employees, which starts with compatibility of emotions with the expectations of the institute. The teachers in [20] study raise the same concern as the voice of teachers in the new reformations are not given credit and their experiences and skills are not taken into account. In such a situation where the habitus has been formed over history through practices, dispositions, and norms [61], gaining emotional capital is possible, but it is still confined by the rules. In spite of habitus being dynamic and shaped through its own possibilities, and not totally structured [33], institutional norms are strong enough to deter attainment of emotional capital. To illustrate, Songul does her best to reflect on her surroundings, and knows well that she is being obedient, and is willing to accumulate positive feelings, which is a rather successful experience. However, there exist structures and norms, in other words, ‘historical classificatory schemes of value’ [62, p.75] formulated throughout years which have made the disposition obedience to the demands of the norm in spite of the detrimental value to the teachers (e.g. [60–63]).

The teachers of this study revealed situations where they managed to build capital and benefit from it. The findings show that Eda manages to have her feelings under control and cruise through the sea of emotions with success, although she might be viewed as non-aligned in the eyes of her students. This is endorsed by [64] political approach, which sees one’s ideology and power relations as important in creating a strong identity for the teacher. Eda’s awareness of her identity makes her more powerful in harnessing emotions for the better. As stipulated by the concept of capital as something constructively shaped [6], it can be asserted that Eda’s ability to turn her emotional labor into capital stems from her qualifications as well as her experience in teaching [18]. This finding lends support to the study by [65] who showed teachers’ past experience along with contextual variable and power relations in the institution shape the identity of the teacher. An educational background in teaching English has also been reported to contribute to the investment of teachers in a community to regulate their emotions in emotionally labored situations at work [66].

It was also found that one of the teachers could benefit from her emotional control at time of interaction with her students and incrementally enrich her social capital. When engaging in a conversation with students, mostly to deal with students’ disorderly conduct or transgression, Songul manages to establish a healthier relationship with her students. She manages her emotions reflectively and finds the best way to win their hearts. Such a conversion to social and cultural capital has been reported in other studies where affective relationships boost the social and cultural capital of teachers [11–39].

Another similar term to emotional labor used in previous studies is emotion management [67]. It entails leveraging the inherent worth of emotions by engaging in a reciprocal exchange of emotions to reap their benefits, according to [7]’s explanation. This means the exchange of emotions for the pure expression of love at school is not emotional labor, but emotion management. However, emotional labor is ‘sold for a wage and therefore has exchange value’ (p. 7). To clarify, some researchers may not see the control of anger in the classroom as emotional labor, but simply emotion work. For them, emotional labor is just one’s surrender to feeling rules [67]. We argue that these concepts can be affected by the individual’s agentive power. We found that Meryem and Songul are working in relatively similar conditions where they are prone to emotionally tense instances due to the disruptive behavior of students. While Meryem retreats to a surrendered position by distancing from her true feelings, Songul actively tries to manage herself, muse about the incident and talks it through with someone to gather some experience from each incident. We propose that in seemingly similar emotionally charged situations, it is the agentive power of individuals that determines the nature of the encounter. Affective states are the result of both micro and macro factors [68, 69]. Micro factors refer to the individuality and agentive power of the teacher, which is the result of their beliefs formed through their educational, social, and cultural background. In equal measure, social, cultural, political and historical states are macro factors that shape our emotional states. This approves the criticism levied against Bourdieu’s conceptualization of capital where the agentive power of the individual is ignored [70]. Since capital is reflected in habitus, which is a combination of a person’s expectations affected by their socialization, we conclude that it is not a straightforward task to categorize the emotional moments of the teacher into capital or non-capital ones. Further studies need to have a deeper analysis of individuals and trace their emotional work over a longer period of time to generate patterns, so that it can be asserted with stronger certainty the labor or capital nature of an incident involving emotions.

### Implications of the study

The main implication of the study is the importance of having our teachers think about their emotional experiences. Reflective practices of teachers strengthen their affective dimension and boost their emotional capital [31]. The mechanism is explained under the concept of ‘feeling one’s emotional state’ [71, p. 133]. [71] explains how consciousness can be applied in encounters where feelings are involved. By feeling, he means going beyond immediate reaction and generalizing an emotionally induced state into similar situations and predicting the presence of an emotional reaction in a given situation. In future encounters, the person could approach a similar situation with knowledge of their emotional vulnerability. Such reflective practices, in [71]’s terms, offer an individual the ability to respond flexibly to a situation that has been gained from the history of interaction with the environment. For teachers, reflection means gaining valuable lessons from their daily interactions with students and other role players. [12] proposes some reflective practices, including (auto) biographical storytelling, mentoring and teacher-bonding relationships, and action research. We should add that such reflective activities can be helpful when the outcome is more tangible to the teachers, as it is a way to know their emotions, fight norms, and gain capital out of each occasion. As an example, in order to collect data for the present research, we initially faced the unwillingness of teachers to share their emotional states with a researcher. However, after the course of the study, all three teachers talked positively about the experience of keeping an emotional log since it would ask them to think about their day and then record the event, an activity that takes a considerable amount of reflection. This experience made our teachers attain some basic knowledge about the types of emotions they go through. That lends support to the benefit of keeping a diary as a reflective tool. Having done that over a long period, teachers can have the most commonly invisible daily emotions visible to them. They can pinpoint these emotions, and then work on them- might need getting help from a specialist-and exploit them well by gaining emotional capital.

The other suggestion could be the formation of emotion-sharing communities among teachers in a liberating manner. That is because an occurrence in a situation for a teacher can be a lesson for another teacher, which provides the ground for sharing related emotions. It also gives teachers chances of bonding with each other to act as sources of encouragement and inspiration for each other. Steps, however, should be taken to ensure that the teacher is at ease that their professionality and character are not colored by having their emotions displayed. More importantly, including such events in the curriculum, establishing power relations, and structuring them in a restrictive way might create reluctance to talk about joyful or stressful events in the class. As such, our proposition to form feeling sharing circles is to encourage teachers with commonalities (sharing the same levels, hours, etc.) to take initiatives and organize everything based on their absolute will and shared feelings. Teachers normally love sharing stories of their class happenings, but they can be informed that such activities can be expanded to be more empathic and this can bring occupational as well as personal accomplishments.

Finally, as [33] placed considerable emphasis on the role of education in gaining cultural capital, we can extend this into the area of emotional capital. Simply put, teachers can be trained to channel their desires into less challenging states. This can be achieved by having basic training in emotional labor and strategies to gain emotional capital.

## Conclusion

Our study shows the emotions three teachers of English go through at work, and cases where they have to suppress emotions like the feeling of care to comply either with institutional or social rules or norms. However, there were situations wherein the teacher could have control over her emotions and enjoy the emotional capital acquired. Our study focused on teachers of English at two schools and a university. However, the data could be enriched with stories of emotional labor experienced by those working at primary schools where expressing emotions is a more distinctive feature of the job and teacher’s emotional health has a tangible effect on students. The present study did not focus much on the agentive power of individuals as a telling attribute in defining a person’s emotional success in a given context. Close examination of a participant’s emotional, social, and cultural background can provide a richer understanding of the maneuver of the teacher when faced with an emotionally charged situation. As far as generalizing the findings is concerned, the present study suffers from limited generalizability power. The ideas raised here can hold true in the cases of many school teachers who are bound by rules mostly coming down from the ministry of education, and those who suffer from students’ misbehavior. However, the literature on emotional capital and emotional labor should be enriched by having more studies on teachers of different backgrounds and levels who practice emotional labor and capital in contextually unique settings. As an example, the status of teachers in societies with more restrictive political layouts where teachers should orchestrate their emotions between pure human feelings and those of societal norms can be another area of research for future enthusiasts in the field. Teaching is a profession with a whirlwind of emotions, which necessitates meeting the expectations of many people, displaying certain emotions and suppressing others. Such practice of pretensions can inject negativity further down the teacher’s life and bring about devastating consequences. As such, the emotional health of teachers should always be a topic of discussion in academia. Teaching has stepped well into modern life and is now a ‘lived and palpable experience of desire, pleasure, and pain’ [72, p.369]. Through further research on emotional labor, the pain involved in teaching can be alleviated.

## Supporting information

S1 Appendix Questions to help participants in their diary keeping attempt. (DOCX)

S1 Data Data of the diaries and interviews. (DOCX)
