# Supplementary material for: Emotional labor and emotional capital: An interpretive phenomenological analysis of teachers of English
Source: PLoS One. 2023 Apr 27;18(4):e0283981. doi: 10.1371/journal.pone.0283981 (PMC10138224; doi:10.1371/journal.pone.0283981)
Supplement: S1 Appendix — (DOCX) [file pone.0283981.s001.docx]

# Appendix

# Questions to Help Participants in their diary keeping Attempt

- Among the emotions listed below, which one do you relate to when teaching?
- happiness/joy/frustration
- sadness/grief/disappointment
- anger/irritation/disillusion
- fear/anxiety/guilt
- disgust/despair
- fascination/caring
- pride/love
- wonder/intimacy
- enthusiasm/loss
- boredom/powerlessness
- awe/compassion
- How do you react when you have such a feeling? Do you think that reaction will help your teaching?
- Where do you think your reaction stems from?
- Which feelings did the students have today? What was their reaction?
- Are there feelings that you try to have by doing something regularly in your teaching?
- Any good/bad experience? Any change in a relationship with (a)student(s)?
- Any emotional mood you had on students or you gained from them?
- Did you share any feelings with students?
- How did you deal with the situation?
