# Supplementary material for: Emotional labor and emotional capital: An interpretive phenomenological analysis of teachers of English
Source: PLoS One. 2023 Apr 27;18(4):e0283981. doi: 10.1371/journal.pone.0283981 (PMC10138224; doi:10.1371/journal.pone.0283981)
Supplement: S1 Data — (DOCX) [file pone.0283981.s002.docx]

**S2: Data of the diaries and interviews**

**Eda**

Every Monday is typically the day I plan my week and check previously arranged appointments if any. Commuting takes 1 hr so that really tires me. Also not having another alternative for free transportation to school is unpleasant. There is a ring but it costs 8 TL for one way, which is expensive when you think about the whole month.

After necessary things are done, you are still expected to stay at school and sign for both evening and morning to state your presence. After all, how will you get out of campus again you will need to consider the scarce transportation options:P

Tea fees were increased from 25 to 40 TL. When you think about the rise in Turkey to all goods and services I can get the reason. However, it is disproportionate because we teach in a private university and they get a lot of money and I think teachers can be made happier with small courtesies like paying the tea fee for us. I don't know.

I hate being at school when I am not teaching. I hate being forced to be at school.

Today I am leaving for the new year's holiday. I got my annual leave. But still I get messages from the teams and unfortunately I learned that I am going to substitute for a teacher's lessons for 3 days. I was planning to observe 3 more teachers but in that case I won't be able to do that. I hate taking messages which include subs news. You are unable to plan your week because of these sudden instances. Omicron is spreading and cases started to be seen at our school. I just want to stay at home and teach online. Why can't we just teach like all the other universities??

I had an exam invigilation today and I felt very disorganised again because I was planning to prepare my workshop and do my readings, then I learnt that I need to invigilate an exam like this, which wasn't in our agenda but testing knew that of course. In the afternoon I felt myself not useful or in good mood. I left school and one of the admins sent a message over teams and told me that she'd given feedback to the lesson plan, which I was considering that we would make a discussion over it and I wasn't expecting bubbled comments.

Wednesday is a good day for me, I am free in the morning so I have some time to get prepared for the class in the afternoon. I love my afternoon class but some students especially don't come just because I keep speaking English all the time and they struggle. I see that. But they just prefer not to negotiate but to escape. I am questioning myself in such situations. I try to be consistent and keep a profile based on my teaching philosophy and learners make me think that if I only  translate in the lesson constantly. I have some flexibility of course but I cannot and I think I shouldn't push those limits that much they ask for.

**Interview data (Eda)**

Yes you know I believe the teacher should not be thinking too much about such things… for example why I should be worried that I should pay this much for this and this much for this and it’s not …not one or two ..many things / I know in some universities like government universities it’s not like that.

Actually they’re good students and lots of energy / I know they have to come to English class every day and they have homework that is that is really too much / they’re innocent and I try to understand them / it’s difficult to be exposed to English for many hours in only one day but I can’t do them a favour by speaking English I mean speak Turkish ….and eeee this is actually not a favour / they just get used to it… of course sometimes I … it’s not like never Turkish / sometimes I try to help them by speaking Turkish a bit but not always … but when I talk in Turkish … then they expect it all the time ..

**Meryem**

It was actually a great day and nothing special happened in spite of having a busy day on Tuesdays having lessons from 9 to 4.30. I had many classes and had to stand up for long hours that was a lot of pressure, but I had a pleasant day emotionally. Of course I had to show a serious face and raise my voice at some points, but …. that is part of the job. I am happy that things have got into a routine way and nothing special happens in the classes.

Mondays and Tuesdays are the busiest days of the week having 8 or 9 classes back to back. There is a FAN LISE class whose students are too proud of their knowledge since their level is high. That is why they do not care about the class. For example, today two of them were playing chess in the class. Although I confiscated the chess set, I realized that they had taken it from my desk and started playing again. I had to confiscate it again and never return it. Another one was singing in the class. The whole class was like a zoo. They do not care at all since they’re proficient English speaker. When I step into the class, I do not even consider my presence. I have to tap on the task and shout a lot to get their attention. Today, I felt like there was no authority left for me in the class. In order to solve the problem, I talked with a colleague, and s(he) recommended sending them to the manager’s assistant to pay a visit to the class and make them quiet. Although I really do not care whether they learn something or not, I just want to have their respect. At least, they need to notice my presence. I teach others classes with noisy students, but these ones are exceptionally noisy and disrespectful. To solve the chess issue, I need to talk to the principle to ask students to come down and have a serious talk.

The point is having classes from morning to afternoon nonstop is bearable for me. There are peripheral issues which bother me a lot. For example, our coordinator wants me to find some seminars to students to attend. I am not paid extra for having club sessions, and she wants me to search for seminars by taking my between-semester holiday. I need to motivate students, check their attendance and all these things make me feel really sorry about this scheduling.

Today there was a small mistake in their [English] test paper… the students had talked inappropriately to one of my colleagues who had got offended due to the behaviour of my students…. I then stepped into the class gesturing serious face and told them I don’t care about your proficiency in English, it’s your behaviour which matters. I was really angry but I just wanted to start the lesson. I started the lesson trying to be serious. Later on I wanted to show them a TED talk. As soon as I played the video, there was a half-naked girl shown on the screen. It was not something terrible, but not good for these teenagers. The class burst into laughter and silly comments could be heard here and there… I felt embarrassed and had to bear students’ funny remarks and their sneers. One of them jokingly said so you don’t check the papers, can’t you just check these materials?

As the assistant on the bus, I am in charge of delivering the student to their home safe and healthy. On the bus, the students should take health measure about wearing their mask. There’s a grade 7 student who does not wear his mask properly. I remind him a couple of times. Then he removes his mask shouting I won’t wear it, and don’t talk to me. I told him I was addressing everyone. When getting off, he was saying I don’t want to see you again. The other students supported me by telling off that student. What I did I didn’t know what to do at first really but then what what I did was to ask the driver to contact his parents and talk about this important issue since the others are too sensitive about that. Well the virus is still a threat I think. That might cause a problem for us. Before we get off the bus, our responsibilities are still there and it’s never ending. Unfortunately, we are in charge of such things.

**Interview data (Meryem)**

I think the problem is not teacher or students, but the system. I mean students are good at English so they should have another lesson. They’re already advanced level. When they come to the class and have four hours with one teacher, so they get bored and start crating problems for the teacher. Usually the night before these classes I am not in mood when I think about having them tomorrow. I don’t understand the system that why there is still English class for them when actually they don’t need any English anymore. The time should be spent on other lesson. Sometimes in my class they start studying for other lesson or doing some other homework. That’s not something I can tolerate. I talk with the principal but they say do not let them to do that. And that’s it.

What could i do? At first i was thinking of going to her and have a talk. But then is it really effective? I thought for a while and then decided not to talk to her and just follow it.

Because you know there’s something in our school that i don’t understand and is very silly I’m sorry but really unreasonable. When somebody gives some even suggestions, everybody says she is doing you know disagreement or rebellion. In our school you are a good teacher or someone who is rebellious , just that. So i thought why should i have this adjective . I know it’s not right but i don’t want to be a rebellious because i might start looking for a job after the term finishes.

Yes about that ted video I was really disappointed when it happened mostly because I had tried to stay serious in the class and did my best to show that they did something very wrong. But then I made a mistake although it was not my mistake but they don’t know. They just think I’ve prepared this video. I felt sorry about their test paper because it was confusing and that should not happen in an important test like this. But at that moment I really didn’t know what should be the important matter here. Should I take side with students because of their test, or my colleague or I should be careful of what they have seen in the ted talk. Maybe I should not have cared at all. I don’t know. Maybe it was not that serious and I just took it hard on myself.

Yeah about that I should say it got under my skin really. But then I thought actually this is something I always try to think of when I lose my temper because of them . I thought that come on Meryem he’s just a child of ten years old or even less. You can’t let him make you angry. You know they are both young and a different generation. So I always think about this and it makes me calm.

**Songul**

What happened yesterday was that when I stepped into class, I have a very very good class. Their level of English is really good. Now I wanted to start the lesson immediately since I was running behind, and I told them / but since they’re so impulsive and too active that I can’t find enough time to cover everything. Besides that, suddenly yesterday they started complaining that we’re not motivated since the principal promised two years ago to have music class, art class and such and such. Why just math is important in this school. we need art and music and he hasn’t kept his promise. So here what happens is that I need to soften the issue although I am aware that they have the complete right. eee actually I had to do three things. First, I should not say something which could you know damage the reputation of Mr … (the boss), second is that I should say something so as not give the boss an excuse to blame me and I also say something to the students so they could trust me and not regret sharing this concern with me. Besides, it’s my humanitarian responsibility. It’s an ethical act to empathize with them and add something here. What I said was to tell them to have a talk with him (the boss) but they didn’t accept because they said we’ve talked with him many times and no use. Then I told them well since you individually go to him and do not get a proper reaction that means you should do something as a group. I mean when you go there one by one then you hear a no more easily but if you go all together at least you’ll drive his attention to this issue.

In the morning I checked the program of the day it was like always  so we had 8 classes and the last 2 hours was our preparation time. Today we had online lessons due to icy roads. What got on my nerve was a message from the boss who said today you will join a link in the last 2 hours. Why was this painful?! During the week we have almost 40 classes and now that we have got a chance to be away from the desk for only 80 minutes, we're deprived of it. I found it insulting and nerve-racking. We cannot react to this as we are" yes sir, boss" at work. So i had to swallow it and let it be like ever. Of course meetings can be good especially for some matters it can be really good. But they should change the time of these meetings , not at the end of the day after lots of hard work and exhaustion. You can’t show any energy in these situations. Worse is that I have to show this energy because I am the longest serving English teacher in this school so I have to comment and ask questions to show that I am happy here and there’s a good relationship. So here meetings are not just to discuss something but to show that everything is perfect.

Knowing the last hours is boring for 11 graders (who are demotivated as their English score doesn’t play any role in their future studies), i decided to show a documentary related to the lesson. But the video projector didn’t work, so i changed the plan. The thing is i am experienced enough to know what to do and how to deal with unanticipated problems, but sometimes it is frustrating because you have to shift from situation to another fast after many ups and downs in a day.

Another thing that was annoying was when students would come to the board and start drawing something on the board. I want to tell them to sit, but I see they’ve drawn a heart with my name in it. this causes the class to get out of control. I try to ask some questions about love and then ask them to sit.

The classroom where the class is held changes almost every day. They don’t change the class but just change the class number. I mean, I should check it every day. And there’s no educational reason behind that unfortunately. For example, if I have classroom number five now , next session it might be 6. And this has not true reason. Apparently they just do it so that students won’t get used to one place, but it makes me agitated to have another burden of keeping track of class numbers. For example, I should always consider this that my class should be big enough to have seats for all 17 students. Or this factor should be cared for that for the 6 grade group, the class should be large enough so there would be enough space for everyone to go around. Another thing is that I should be in a class with the video projector since they are 5 and 6 graders so they learn better if they see things on the screen and I can’t stick to the book only. But they don’t think about such matters.

Something extraordinary happened today. We wanted to go to the class, the after lunch class. I have this class with 17 naughty 5 and 6 graders. I wanted to enter the class but I saw that the principal was in my class. He’s seen something from behind the window and was yelling at them because of their misbehavior. So everyone was blaming each other and some would say it was not me or it was him or her such and such. Then he (the principal) made all 5 graders make a line in the sport salon. Then he asked teachers to be there too . So when we went there we saw that everyone was there standing in a line. He then started swearing and yelling . it was so loud that everyone was shocked. So ok I can understand. They’ve probably misbehaved. Then he told them that from now on till 6.30 , just imagine they’re 5 year olds who cannot stand still for even a minute. He says you have to stay from 1.30 to 6.30 in sport salon , stand up with no talking, walking or anything. He locked the door like having prisoners. He had them stand up till 3.30 and the students were feeling exhausted. They were not allowed to go out , eat or anything. Then they could take a chair, sit and do nothing for the remaining time. We had a colleague who got really angry at this and said I imagine that this is being done to my child. So he starts complaining and saying he wants to confront the principal. Something that was a bit scary was that I had to have some serious talks with her to calm him down and stop him from doing anything stupid. That might cause a tension and of course he was not listening. He really wanted to have some sort of fight here. He then grabs a chair and sits with students without doing anything. The principal sees this scene, is shocked a bit , so the guy assumed that he was having a sit-in. Our colleague (the sit-in guy) was furious and that made us upset. Then we had a discussion whether this was right or wrong. I would always say that we’re not confronting one person only. What can we have to present in these situations, I cannot interfere. It’s what the community wants. It’s what their parents want. It’s not like going and saving the kids. If I want to jump in, I might face problems. A teacher can’t save the kids. I believe it’s just the school culture. I won’t be able to do anything. I’d rather walk away. It is not just one person. It is the dominant ideology. I personally think this is something cultural and I really can’t do anything here.

The students’ hugging me has created some problems for me. There are a couple of students who do not let go of me when they start hugging. It takes something like 2 minutes or so to loosen up. They keep going and hugging harder for a couple of minutes. They’re two boys, five graders. I try to tell them to stop and sit down, but they press on and sometimes it gets worse. The problem is that it is too much, and it takes a lot of time. I can’t behave harshly here; they’re just kids showing sentiments. I try to convince them with jokes and a lot of smiles to make them get back to their seats, though they do it again from time to time. I don’t have a good feeling here because people might see us from the windows and me being hugged by two boys all the time I think is not a good scene.

Something that happened today was that I had a class with kids. Something I learned today was that shouting and yelling at these small kids is not that effective, but applying some sense of humor, now they like me and I love them too. Although I am never ever into teaching kids, I have managed to handle these 17 children. Well I am doing my best. Today I was not feeling well , had a headache and stomach ache, but I had to have fun with them, make jokes and do some laughter. The point is in their class, I am not just a teacher of English. I should be their friend, their councillor.

Yesterday I had a chaotic class. Students love coming to the board without asking permission and they start drawing something. It just happens out of sudden and I kinda of lose control of the class. I want to ask them to sit, but then I see a heart with my name in it. It’s nice but they should sit because it’s just killing time. But I can’t get angry at them. I try to copy their drawing, ask some questions about love, and then demand they sit. It has worked so far.

Yesterday there was this kid sitting in the first row. Of course they’d already been punished the other day when they had to stand still for hours. Anyways he suddenly slaps his friend next to him of course he was playing a prank on him. Then I got really angry. I asked him to go out. He says I’m sorry hocam, and I say no every session you say the same thing. You should go now. He says I was just playing a prank, it was not serious. I go like yeah I know you were joking and it was not serious. Then the guy next to him says hocam it was just a joke such and such. I said ok if it was a joke, I’m also your friend, so I can slap you right? They said no no it’s not something good. Then I think he learned his lesson. If he does it again, it means he’s probably used to it.

**Interview data (Songul)**

Well what can I say. It was a stressful situation for me for I can’t find enough time to finish the lesson. You know we have a book that has twelve units or ten. Yeah twelve units plus some other extra materials like a story book and a grammar book. Sometime the class hour is taken for a quiz or another activity. So I don’t have enough time . and students of course they don’t care because they are young and if you play a game with them every session, they’re always happy. So I should try to find time to teach. Then they come to the board and start hugging. I think everyone loves hugging, but it’s not right. Why isn’t it right. Because first it takes a lot of my time actually their own time. second it’s not nice in this society. we’re living and working in turkey. It’s not the united states. If someone sees us, of course I know they’re very young and even adults of men and women here sometimes give each hugs but if it happen a lot and every time people see us I think it’s not nice in the eye of the society. it might look not a very serious thing but imagine every day I have these in my classes. Then if our angry principal sees that he thinks I’m always playing with them hugging them kissing them.

I consider myself as an experienced teacher but this experience has been with adults so far. So I had problems with kids. They’re nice and cute and I love them. Well I think everybody loves young kids. But when it comes to teaching them it’s a different story. They’re noisy and misbehave most of the time. Yeah but I try to think about ways to control them because when I have classes with adults I always think about how I can be a better teacher. Of course I love just yelling at them and have the class quiet for a few minutes but … . You know it engages my mind so much that I sometimes share it with …. (her husband). We talk about my day and he gives me some solutions. Of course sometimes his solutions are a bit harsh but it’s good talking with him about my job. So I learned that with children you can’t always yell . Maybe they become quiet for a few minutes but then they start again. So befriending them is a better option. I think just my experience helped me find solution for such a problem. Still I’m learning actually. When I go to the class and something happens and usually something happens, I just try to find ways to improve it you know.
